# Calibration of a climate suitability model using a generalized likelihood uncertainty estimation (GLUE): a global case study of orange production

**DOI:** 10.1038/s41598-026-44664-5

**Published:** 2026-04-17

**Authors:** Shinwoo Hyun, Kwang Soo Kim, Robert M. Beresford

**Affiliations:** 1 Research Institute of Agriculture and Life Sciences, Seoul National University, Seoul, Republic of Korea; 2 Department of Smart Systems, Seoul National University, Seoul, Republic of Korea; 3 New Zealand Institute for Bioeconomy Science Limited, Private Bag 92 169, Auckland, 1142 New Zealand

**Keywords:** Species distribution model, Likelihood, EcoCrop, Presence-only data, GLUE, Climate sciences, Ecology, Ecology, Environmental sciences

## Abstract

Parameter calibration of climate suitability models is often hindered by the lack of absence data for plant species, limiting their effectiveness for global scale applications. Here we propose a novel calibration approach based on the Generalized Likelihood Uncertainty Estimation (GLUE) framework that eliminates the needs for background samples. This method defines the likelihood statistics under the assumption that the distribution of the climate suitability index at the occurrence sites differs from that across all locations within a given region. To prioritize presence data, a weighted likelihood function was incorporated into the GLUE procedure. We demonstrated the utility of this approach through a case study on orange (*Citrus sinensis*), a crop whose climate suitability has rarely been evaluated at a global scale. Model performance improved when the parameter search spaces were defined with minimal ecological constraints, which resulted in a clear separation between producing and non-producing countries. These findings suggest that the proposed approach offers a robust and scalable alternative for climate suitability modeling in data-sparse contexts. This framework is broadly applicable to both cultivated and invasive species, enabling reliable projection of the potential distribution to inform land-use planning and climate adaptation strategies.

**Supplementary Information:**

The online version contains supplementary material available at 10.1038/s41598-026-44664-5.

## Introduction

Strategic reallocation of crop production areas has been identified as a critical adaptation measure for reducing climate change–induced risks to agricultural systems^1,2^. Malhotra^3^ and Conradi et al.^4^ suggested the phytoclimatic conditions such that the ranges of temperature and precipitation occurred within areas where the adequate duration of crop growth would be met without abiotic stresses such as heat, cold, and water stresses. Haokip et al.^5^ suggested that the assessment of bioclimatic boundaries would reveal the regions where crop yield and quality can be maintained or even improved under future climate conditions.

Species distribution models (SDM) have been used to determine the potential boundary of crop production areas at regional and global scales using climate data^6^. For example, MaxEnt model has been successfully used to predict the spatial distribution of crops in a region of interest^7^. It predicts the probability distribution of a species using the presence-only data as inputs to achieve the maximum entropy^8^, which allows for greater reliability of the model than other SDMs. Still, Fitzpatrick et al.^9^ reported that it may overestimate the spatial extent of species distribution. Such drawbacks often resulted from the biases of observed occurrence records, which would violate the assumption of an independent sampling from the species’ underlying spatial distribution^10^. The use of MaxEnt model could also be limited to the regional scale because its assumption may not be met due to human-mediated introduction, socio-economic factors and selection of background sample from heterogeneous environmental conditions.

Alternatively, climate suitability models have been used for crops using knowledge of ecological properties rather than machine learning of background samples as well as presence-only data^11–13^. In assessment of climate suitability, the linguistic rules have been evaluated using the fuzzy logic operations of climate conditions for different crops at a global scale^14^. The parameter values of these models were often obtained from existing literature or the EcoCrop database (https://gaez.fao.org/pages/ecocrop), which provides records on environmental conditions for more than 2400 crop species. Ramirez-Villegas et al.^12^ and Pourmeidani et al.^2^ assessed the climate envelopes of sorghum and medicinal plants using the parameters obtained from the database. Still, Møller et al.^15^ reported that the parameter values available from the EcoCrop database were not suitable for apples in Denmark. Such findings highlight the need for parameter calibration of the model for the crops of interest^16–18^.

Different approaches such as Simplex and Metropolis Hastings algorithm have been used to calibrate the parameters of crop models^19,20^. Nevertheless, it would be challenging to apply these conventional methods to a climate suitability model. The objective function for the existing algorithm is often defined to minimize the difference between observation data and the outputs of the given model. Such an approach would require both presence and absence data in order to evaluate the degree of agreement for the objective functions. However, the need for the latter poses a challenge due to the scarcity of reliable absence data^8^.

Royle et al.^21^ suggested that a Bayesian approach would be applicable to the evaluation of spatial distribution models. This approach would assume that the suitability of climate conditions would be high at all the occurrence sites. Kim et al.^14^ derived a joint likelihood function based on such an assumption to compare the climate suitability models using Bayes’ rule. This suggested that the likelihood function can be used as an objective function for parameter calibration of climate suitability models.

The parameter calibration procedure often starts with the definition of the prior distribution of each parameter^22^. A uniform distribution within a specific range is typically used to define the parameter space^23–25^. The minimum and maximum bounds for parameter values in the search space can be determined for the climate suitability model using knowledge on the plant species of interest. Still, such ranges could affect the reliability and efficiency of the calibration due to the sensitivity of the model to the parameters^26^. Although climate envelope is available from existing databases, its reliability may vary among species, leading to uncertainty in the parameter ranges for calibration.

Here we propose the Generalized Likelihood Uncertainty Estimation (GLUE) framework with a weighted likelihood function as an approach to calibrate the climate suitability model using the presence-only data. In particular, we focused on the development of an alternative method for calculation of likelihood statistics that quantifies differences between the distributions of suitability values at species occurrence locations and those across the full spatial domain, especially at a global scale. By relying on distributional contrasts rather than explicit background samples, this framework reduces dependence on random generation of absence data. Such an approach is based on the assumption that species occurrence is constrained to regions defined by specific climatic conditions, enabling model calibration without predefined background sampling at the global scale. We also investigated the impact of the search space boundaries for the parameter calibration. The research questions of this study were as follows: (1) what is the difference between existing and alternative indicators to evaluate likelihood for assessment of climate suitability? (2) what is the impact of the range of parameter values derived from the existing knowledge on the outcome of calibration? and (3) what is the improvement in the assessment of the spatial distribution of a crop of interest using the new calibration indicator?

A case study was performed to answer these questions on parameter calibration of the climate suitability model for orange (*Citrus sinensis* (L.) Osbeck), which is one of the most popular and widely consumed fruits around the world^27^. By applying the fuzzy logic model to *C. sinensis* at a global scale, we demonstrate how this framework can reasonably delineate the spatial distribution of a popular citrus crop. This work aims to demonstrate the practical utility of combining presence-only calibration and fuzzy rule-based modeling to improve prediction of the spatial distribution for the given crop.

## Theoretical approaches

### Fuzzy union model

Fuzzy Union model, which is based on t-conorm or fuzzy union logical operation, determines the climate suitability index using monthly climate data as inputs^14^ (see supplementary information 1). This model estimates climate suitability by maximizing suitability across different start times of growing period. For each start time, climate suitability is evaluated for multiple possible growing period durations, and the median value across these durations is selected.

**Fig. 1:**
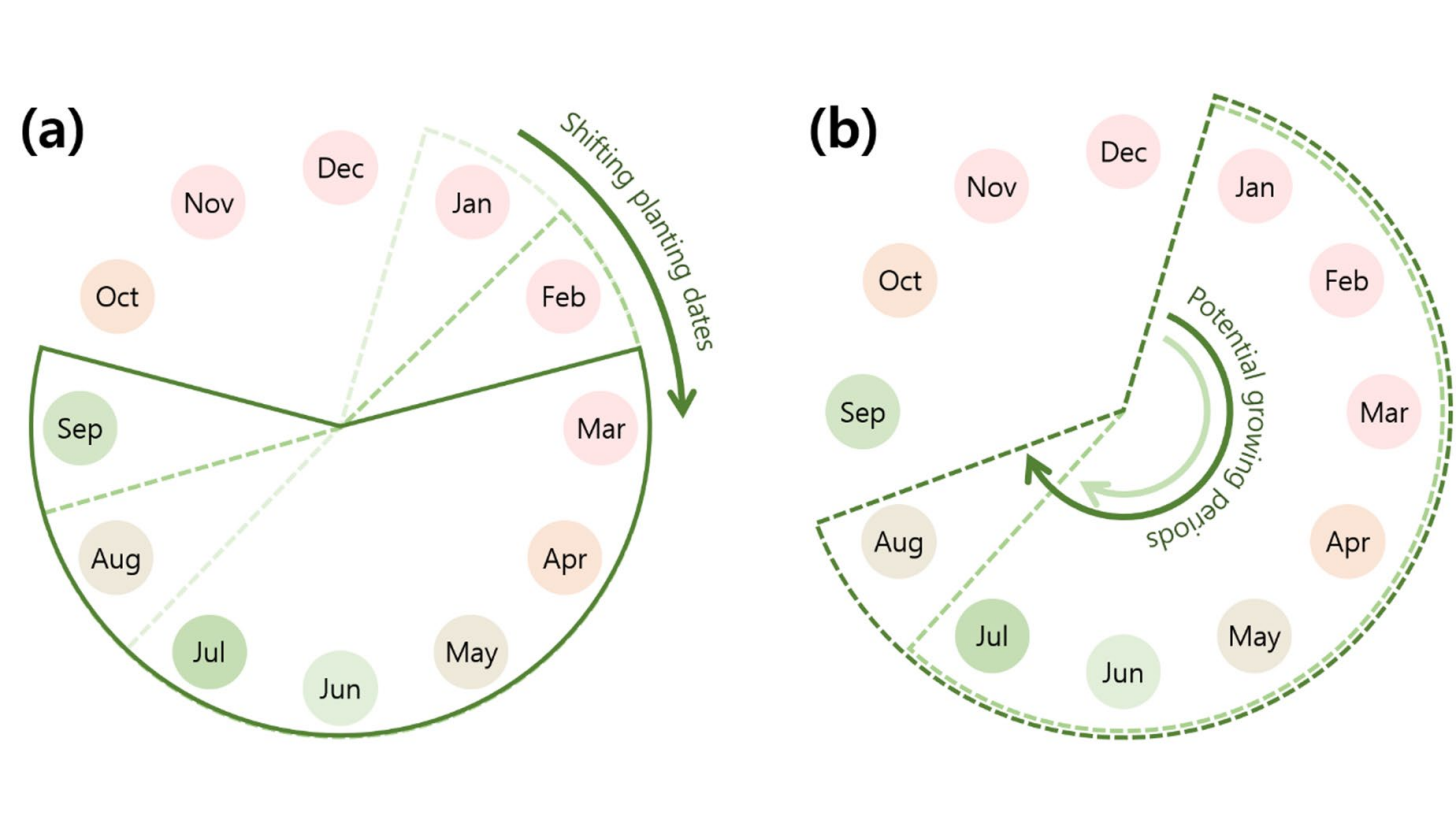
Illustration of calculation procedure for the climate suitability index with shifting **a** growing seasons and **b** growing periods within the growing season using the Fuzzy Union model. A growing season is identified by a planting date. The final climate suitability index is set to be the maximum value of suitability index across different planting dates. The suitability index for a given growing season is determined by taking the minimum suitability value across the growing period durations. The suitability index of each growing period is determined by averaging the monthly suitability within the given period. The color of each circle indicates the climate suitability index in a given month.

The aggregation algorithm used in the original model represents a management strategy to reduce the risk of crop failure, although it may not maximize crop yield. This approach can be applied to annual crops for which farmers can adjust planting and harvest timing. Such an assumption is not valid for fruit trees whose growing period and seasonal exposure are fixed and cannot be altered by growers. Therefore, in the present study, the Fuzzy Union model was modified to determine the climate suitability index for orange trees using the minimum suitability value across the growing period durations (Fig. 1). In addition, the model was also modified to incorporate winter survivor, as evaluating suitability based solely on the growing period may underestimate climatic risk for perennial crops that remain in the field over the entire year.

### The generalized likelihood uncertainty estimation

The GLUE is one of the global optimization methods to calibrate model parameters^25^, which explores the entire parameter space to find the global optimum^24^. The GLUE method has been used in a wide range of studies due to the flexibility of the likelihood function as well as the simplicity and ease of implementation^28^. In the GLUE method, the posterior distributions are generated using the prior distribution and the probability for each parameter^17^. The parameter values are determined using the mean value of the posterior distribution^25^. In the present study, the GLUE method was implemented for the climate suitability model (see supplementary information 2).

### Likelihood functions of GLUE

Houska et al.^29^ suggested that the GLUE method has flexibility in the choice of likelihood functions, enabling the use of alternative measures that may better suit specific modeling contexts. The likelihood statistics represent the degree of similarity between the observation data and the model outputs obtained using a given set of parameters. In this study, the observation data correspond to the distribution of occurrence sites, whereas the model outputs represent the distribution of suitability values. As a result, parameter sets producing smaller errors would have greater likelihood values, which would result in a higher posterior probability^28^. Still, lack of absence data would make it challenging to use the degree of agreement statistics such as true positive rate and false positive rate in order to calibrate the climate suitability model.

Kim et al.^14^ suggested using a likelihood function (*L*_*kim*_) to compare the outcome of the climate suitability model with the occurrence data of a species. The value of *L*_*kim*_ is determined using the discretized values of climate suitability index as follows:

1 $$\begin{array}{*{20}c} {L_{{Kim}} = \frac{{E_{o} \left( 0 \right)}}{{E_{a} \left( 0 \right)}} \cdot \prod _{{i = 1}}^{{100}} \frac{{E_{o} \left( i \right) - E_{o} \left( {i - 1} \right)}}{{E_{a} \left( i \right) - E_{a} \left( {i - 1} \right)}}} \\ \end{array}$$

where *E*_*o*_*(i)* and *E*_*a*_*(i)* represent empirical cumulative density functions obtained from the occurrence sites and the entire region of interest for the suitability value *i*, respectively. Equation 1 can be rewritten to determine a log-likelihood value (*LL*_*Kim*_) as follows:

2 $$\begin{array}{c}{LL}_{Kim}=log\frac{{E}_{o}\left(0\right)}{{E}_{a}\left(0\right)}+{\sum}_{i=1}^{100}log\frac{{E}_{o}\left(i\right)-{E}_{o}\left(i-1\right)}{{E}_{a}\left(i\right)-{E}_{a}\left(i-1\right)}\end{array}$$

**Fig. 2:**
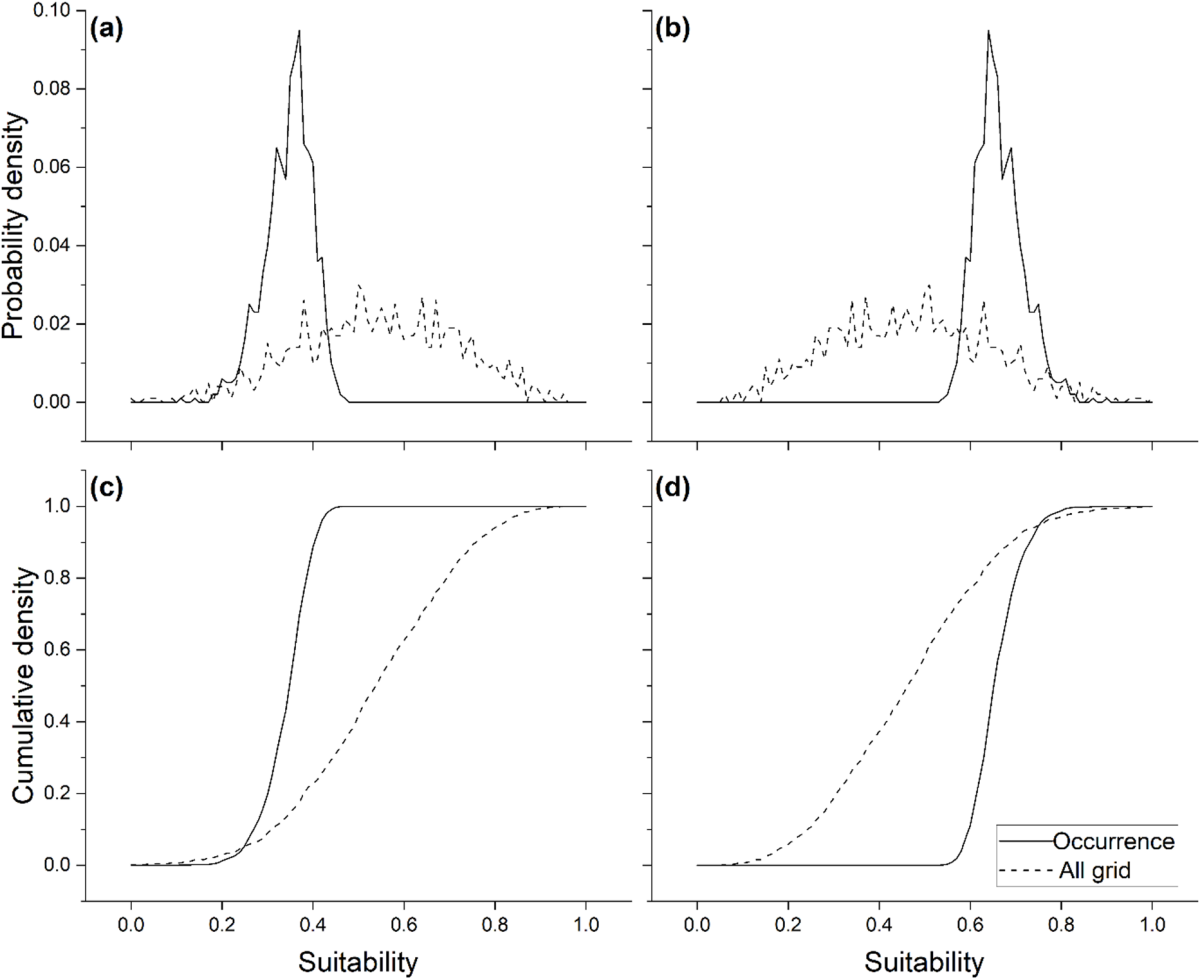
Distributions of arbitrary suitability index (**a** and **b**) and their empirical cumulative density functions (**c** and **d**) at the occurrence sites and within the entire regions of interest. The suitability index values were randomly generated using a Weibull distribution function. The average of the suitability index at the occurrence sites is lower than that of the entire region (**a** and **c**) and vice versa (**b** and **d**).

The log-likelihood value determined using Eq. 2 would be high when the suitability values are high only at the occurrence sites (Fig. 2). However, it is also possible that Eq. 2 could result in a high likelihood value when the suitability index values are high at a large number of sites, even though the index values are low at occurrence sites due to unreliable parameter values. For example, the value of *LL*_*kim*_ would be similar under a scenario where the distribution of the suitability values has the probability density functions (see supplementary information 3).

Hu and Zidek^30^ proposed a weighted likelihood framework that reflects differences in the importance of specific data. In the present study, this weighting scheme was introduced to reduce the impact of less reliable likelihood contributions during the calibration process. In particular, the weights were determined to increase with suitability, with the constraint that the sum of all weights equals one, as follows:

3 $$\begin{array}{c}{w}_{i}=\frac{i}{{\sum}_{j=1}^{100}j}\end{array}$$

where ${w}_{i}$ represent the weight for a discretized suitability index *i*. A weighted log-likelihood (*LL*_*w*_) was defined for *i* as follows:

4 $$\begin{array}{c}L{L}_{w}={\sum}_{i=1}^{100}log\left(\frac{{E}_{o}\left(i\right)-{E}_{o}\left(i-1\right)}{{E}_{a}\left(i\right)-{E}_{a}\left(i-1\right)}\right)\cdot{w}_{i}\end{array}$$

### Evaluation of likelihood functions

The likelihood functions were evaluated using the distribution of suitability index values that could be obtained in a region of interest (Fig. 2). A probability density function was used to sample the probable suitability index values at occurrence and non-occurrence sites, which were denoted by *S*_*occ*_ and *S*_*non*_, respectively. These values were pooled to create a dataset for the suitability index at all the sites *S*_*all*_ under the scenarios of both reliable and unreliable parameter sets during the parameter calibration. The outcomes of the likelihood functions were compared to examine which function could be used to distinguish the parameter set for reliable estimation of climate suitability.

**Fig. 3:**
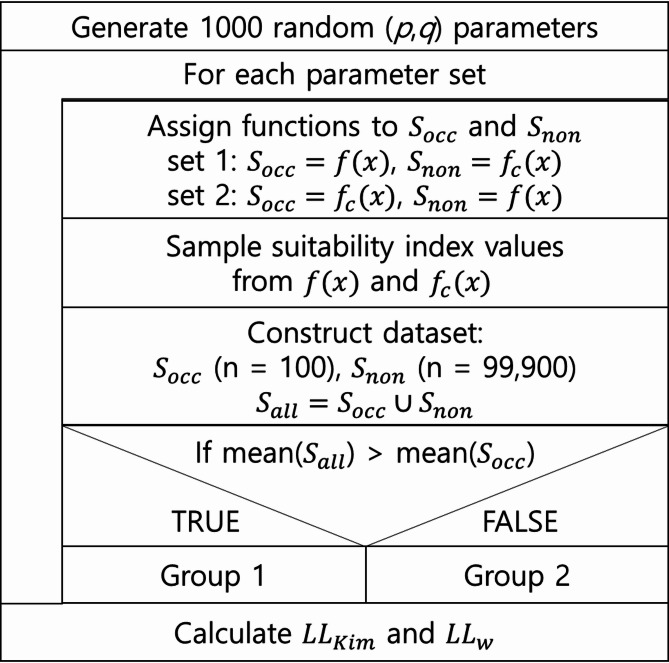
Overview of synthetic data generation and likelihood calculation. *f(x)* and *f*_*c*_*(x)* represent the Weibull distributions and its complementary distributions, respectively, used to generate synthetic datasets. *S*_*occ*_ and *S*_*non*_ represent the suitability values at occurrence and absence sites, respectively.

The Weibull distribution, which has been used in various fields of ecology^31^, was applied to generate suitability index values that could be obtained during the parameter calibration of the climate suitability model (Fig. 3). It was assumed that the index values *x* had the skewed distribution defined by the Weibull distribution function *f* as follows^32^:

5 $$\begin{array}{c}f\left(x\right)=\frac{q}{p}{\left(\frac{x}{p}\right)}^{q-1}{exp}\left\{-{\left(\frac{x}{p}\right)}^{q}\right\}\end{array}$$

where *p* and *q* are scale and shape parameters, respectively. In the present study, the ranges of *p* and *q* were set to be between 0 and 1, and between 0 and 10, respectively. Another set of the index values was sampled from the complementary distribution *f*_*c*_ using the given values of *p* and *q* as follows:

6 $$\begin{array}{c}{f}_{c}\left(x\right)=f\left(1-x\right)\end{array}$$

The generation of the index values using both Eqs. 5 and 6 allowed for efficient representation of reliable and unreliable outcomes of the parameter calibration. When the suitability index values were sampled out of a range between 0 and 1, these values were set to be 0 or 1, depending on the magnitude of the random values.

In each set of random generation, 100,000 index values were sampled to represent the values of *S*_*all*_. These values consisted of 100 and 99,900 samples that represented the values of *S*_*occ*_ and *S*_*non*_, respectively. For each parameter pair (*p*,* q*), two different distributions of suitability values were constructed by alternately assigning the functions *f(x)* and *f*_*c*_*(x)* to *S*_*occ*_ and *S*_*non*_, i.e. *S*_*occ*_ from *f(x)* with *S*_*non*_ from *f*_*c*_*(x)*, and vice versa. In total, 1,000 pairs of parameters for the Weibull distribution were used to create 2,000 distributions of suitability index values (Fig. 3). *R* (version 4.2.1), which is an open source computing environment for statistical analysis^33^, was used for random sampling and data analysis for the suitability values. For example, *rweibull* function was used to sample random values from the Weibull distribution function.

Random datasets of the suitability index values were divided into two groups, depending on the mean values of *S*_*all*_ and *S*_*occ*_ for a given distribution. Once reasonable parameter values were obtained from the calibration processes, *S*_*occ*_ would have a left skewed distribution whereas *S*_*all*_ would have a right skewed distribution because the occurrence sites would locate to areas with a small range of climate envelopes suitable for a crop. The datasets where the mean value of *S*_*all*_ was greater than that of *S*_*occ*_ were classified into Group 1 and otherwise into Group 2. For each dataset, the log-likelihood values were calculated using Eqs. 2 and 4. These values of log-likelihood were compared between the groups and the likelihood calculation methods. In the present study, the Wilcoxon rank sum test was performed to compare the log-likelihood values between the groups^34^.

## Case study

### Input data

**Fig. 4:**
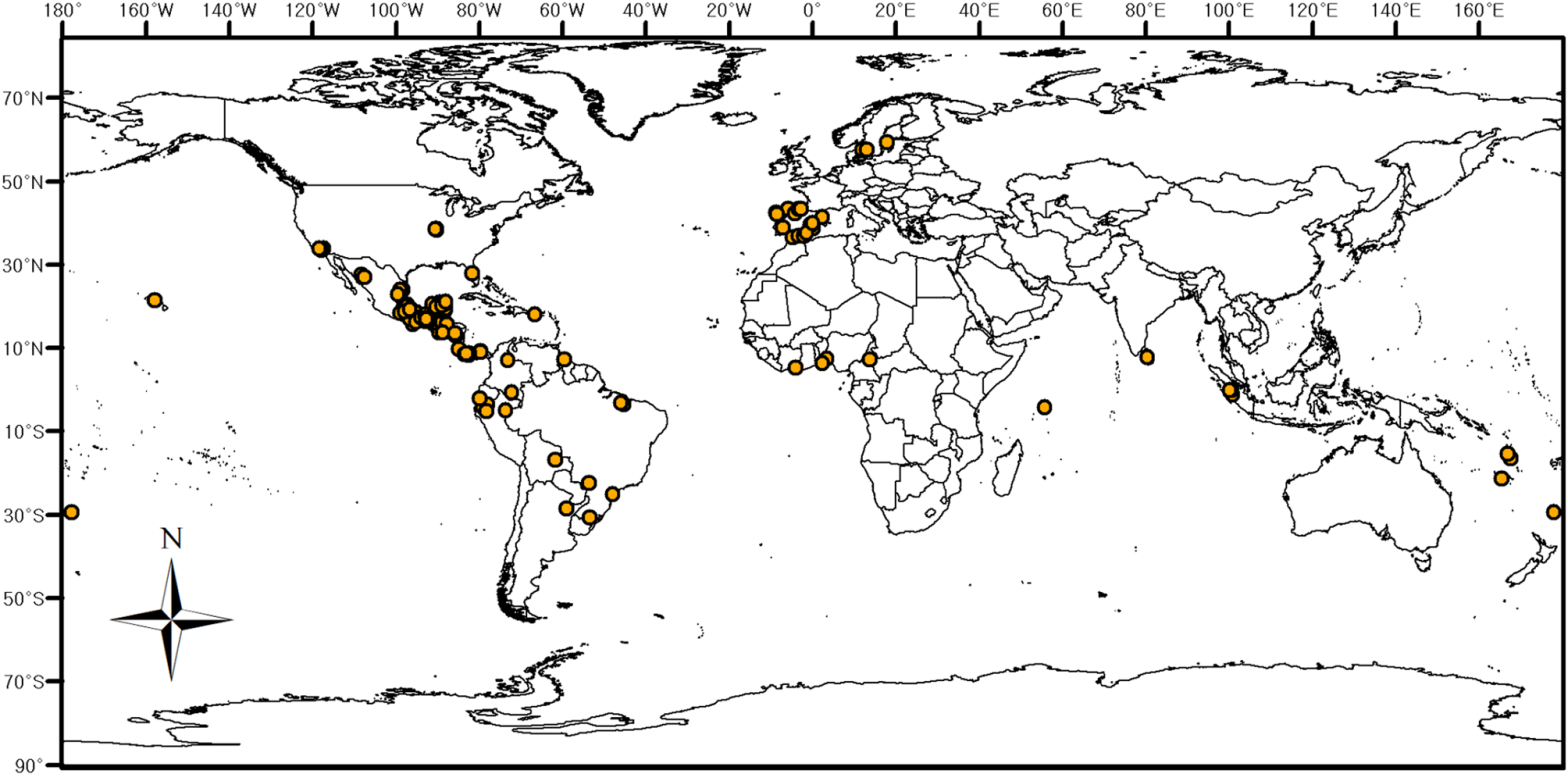
Spatial distribution of the orange occurrence sites, which were obtained from the Global Biodiversity Information Facility and subjected to the subsampling procedure. The map was generated using ArcMap (version 10.5; http://desktop.arcgis.com/en/arcmap/).

The occurrence data for oranges were obtained from Global Biodiversity Information Facility (GBIF), which is the source of biodiversity data open to the public^35^. The data for *C. sinensis* were filtered to include geographic coordinates and year of data collection^36^. A subsampling procedure was performed to make balanced use of the occurrence records^37^. The map of the occurrence sites was divided by grid cells at the resolution of five arc minutes, which is identical to that of climate data. In each grid cell, only a single occurrence site was selected if there were multiple sites. In addition, the occurrence data from 1970 to 2000 were filtered further to match the period of climate data. As a result, 163 occurrence sites were collected for oranges at the global scale (Fig. 4).

Climate data were obtained from the Worldclim database, which provides monthly climate data at the global scale^38^. The database provides minimum temperature, maximum temperature, and precipitation at different spatial resolutions. In the present study, historical climate data for the period of 1970–2000 were used for the calibration. The spatial resolution used as inputs to the climate suitability model for calibration was five arc-minutes, which allowed a reasonably short computing time.

### Calibration of parameters

In the present study, five parameters of temperature were calibrated for the Fuzzy Union model using GLUE (Table 1). The climate suitability model requires 12 parameters related to growing period, temperature, and precipitation conditions to determine the climate suitability index. Still, it would be preferable to calibrate the temperature parameters because temperature suitability was determined using the duration of suitable temperature conditions rather than the range of cardinal temperatures listed in the EcoCrop database. For example, the temperature suitability is assessed using the length of time periods during which a set of optimum temperatures was met in the climate suitability model. In addition, the number of occurrence sites was relatively small for reliable calibration of all the parameters.

**Table 1:** The parameters required by the Fuzzy Union model. The default parameter represents the values from the EcoCrop database.

Name	Description (unit)	Default	Min^a^	Max^a^
*G*_*min*_	Minimum growing period (days)	180	–	–
*G*_*max*_	Maximum growing period (days)	365	–	–
*T*_*killR*_	Killing temperature during rest (℃)	− 10	–	–
*T*_*kill*_	Killing temperature during growing season (℃)	0	− 10	13
*T*_*min*_	Minimum absolute temperature (℃)	13	0	20
*T*_*OPmin*_	Minimum optimal temperature (℃)	20	13	30
*T*_*OPmax*_	Maximum optimal temperature (℃)	30	20	38
*T*_*max*_	Maximum absolute temperature (℃)	38	30	46
*R*_*min*_	Minimum absolute annual rainfall (mm)	450	–	–
*R*_*OPmin*_	Minimum optimal annual rainfall (mm)	1200	–	–
*R*_*OPmax*_	Maximum optimal annual rainfall (mm)	2000	–	–
*R*_*max*_	Maximum absolute annual rainfall (mm)	2700	–	–

**Fig. 5:**
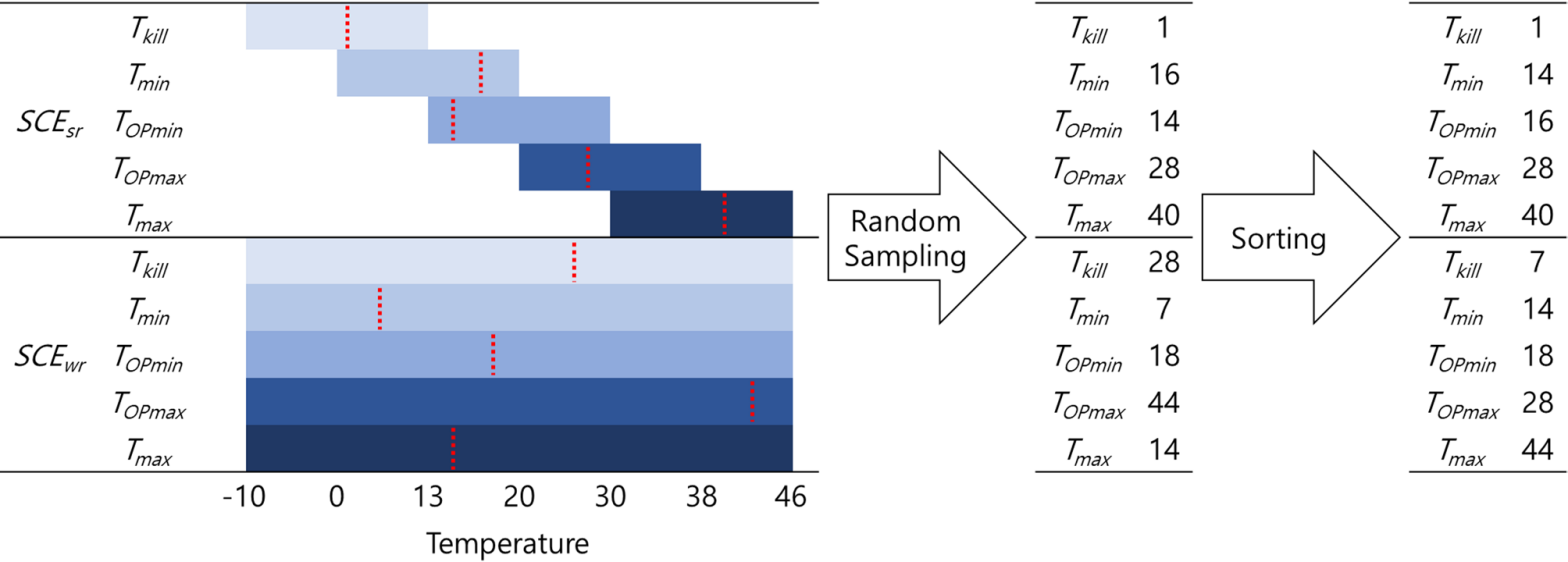
A schematic illustration of random parameter sampling for model calibration. Two sampling scenarios, specific range (*SCE*_*sr*_) and wide range (*SCE*_*wr*_), are used to compare the parameter calibration processes using the Generalized Likelihood Uncertainty Estimation (GLUE) methodology. *T*_*kill*_, *T*_*min*_, *T*_*OPmin*_, *T*_*OPmax*_, *T*_*max*_ indicate killing temperature, minimum absolute temperature, minimum optimum temperature, maximum optimum temperature, maximum absolute temperature, respectively. The dotted lines represent an example of the initial random sampling. These initial values are subsequently rearranged based on their magnitude.

The climate suitability index values were determined using three sets of parameter calibration scenarios. First, the parameter values were obtained from the EcoCrop database to determine the climate suitability indices, which were referred to as default parameter (*SCE*_*d*_). In addition, two alternative calibration scenarios were employed by defining different parameter search spaces, representing contrasting levels of prior knowledge available for the species of interest. For example, the parameter search space was defined using different ranges of temperature parameters from the EcoCrop database (Table 1), which was denoted by *SCE*_*sr*_ and *SCE*_*wr*_ (see supplementary information 4). The parameter values should conform to a logical order before they are used as inputs to the model. The sampled parameter values were reassigned to make sure that they followed a logical order: *T*_*kill*_ < *T*_*min*_ < *T*_*OPmin*_ < *T*_*OPmax*_ < *T*_*max*_. When the values were sampled in an inverted order among the parameters, they were systematically rearranged to meet this biological constraint (Fig. 5).

The GLUE procedure was implemented in *R*. In this study, 100,000 random parameter sets were generated in the first step. The parameter sets were generated based on the Sobol sequence, using the *R* package *randtoolbox*^39^. The Fuzzy Union model was launched using given parameter values to create a map of climate suitability index in raster format. The *terra* package was used to read the climate suitability maps and extract the suitability index values at occurrence sites and all the grid cells^40^.

K-fold cross validation was applied to reduce the risk of sampling bias for the occurrence sites^41^. Occurrence data were randomly split into five subsets, where four of them were allocated for the calibration set and the remaining one for the validation set. Five sets of candidate parameters were generated from the calibration subsets. The parameter set that had the greatest likelihood value for the corresponding validation subset was chosen to be the final parameter set.

The distribution of the likelihood value was examined by parameter for the given calibration set. This would provide an insight on the outcome of parameter calibration under the scenarios based on the range of search space. For example, the likelihood statistics of the parameter sets could vary depending on the values of individual parameters, which would suggest differences in parameter sensitivity and the suitability of the chosen search space.

### Evaluation of suitable areas using the calibrated parameters

Oranges are primarily cultivated in climatically suitable regions, as climate acts as the fundamental limiting factor for their cultivation^42^, although production depends not only on climate suitability but also on socio-economic factors. Based on this assumption, the calibration outcomes under three scenarios were evaluated by comparing suitable areas between countries with and without orange production. Countries with official statistics on orange acreage, as reported by the Food and Agriculture Organization (FAO)^43^, were assumed to be orange-producing countries, whereas countries without such data were considered non-producing.

The comparison was conducted using relative suitable areas (*RA*_*s*_), defined as the proportion of suitable area to the total area within a given country^44^. This accounts for differences in country sizes, as countries with larger land areas are likely to have greater suitable areas regardless of the proportion of climate suitability. Suitable areas were identified as the sum of grid cells with a suitability index greater than a threshold value *TH*, which was defined as the 10th percentile of climate suitability values at occurrence locations^45^. Country boundary information was obtained from the Database of Global Administrative Areas (GADM)^46^.

To compare the distribution of *RA*_*s*_ between orange-producing and non-producing countries, empirical cumulative distribution functions (ECDFs) were generated, and differences between these ECDFs were assessed using the Kolmogorov-Smirnov (KS) test. The *ecdf* and *ks.test* functions in *R* were used for the analysis.

## Results

### Comparison between likelihood calculation methods

**Fig. 6:**
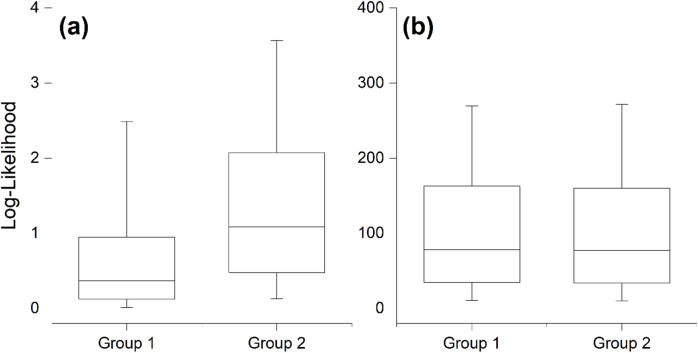
Distributions of likelihood values using **a ***LL*_*w*_ and **b** *LL*_*kim*_. Group 1 represents the datasets with higher mean values of the suitability values at all sites (*S*_*all*_) than the suitability values at occurrence sites (*S*_*occ*_). Group 2 represents the dataset with a higher mean value of *S*_*occ*_ than *S*_*all*_.

The alternative likelihood method showed better performance to assess climate suitability than the conventional likelihood method (Fig. 6). The log-likelihood values for *LL*_*w*_ were significantly higher in Group 2, which represented the distribution of suitability index that could occur in reality, in comparison to Group 1 (*p* < 0.05). For example, the median values of *LL*_*w*_ were 0.4 and 1.1 for Group 1 and 2, respectively. In contrast, the distribution of *LL*_*kim*_ were similar between both groups (*p* > 0.05). The median values were 77.2 and 78.2 for Group 1 and Group 2, respectively.

### Cross validation of climate suitability model

**Fig. 7:**
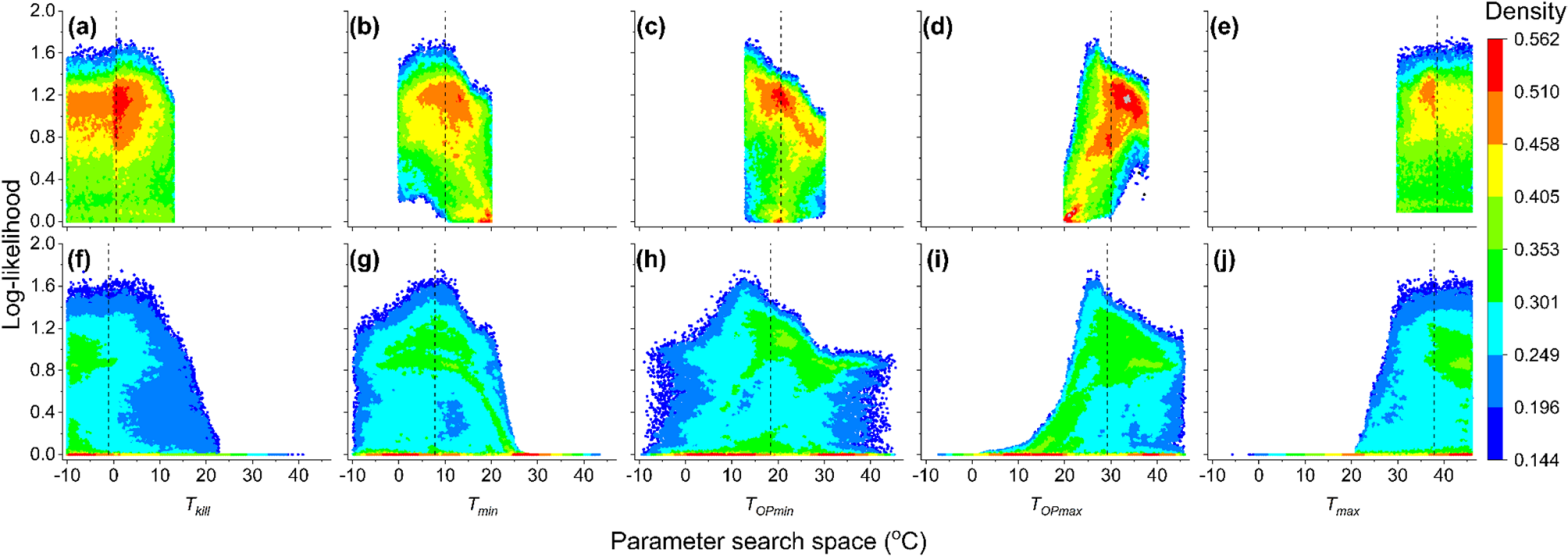
Distribution of weighted log-likelihood values for the parameter sets obtained from the calibration, shown by parameter and search space. Panel **a**–**e** and **f**–**j** represent distributions of weighted log-likelihood values under specific range and wide range calibration scenarios, respectively. Panel pairs **a** and **f**, **b** and **g**, **c** and **h**, **d** and **i**, and **e** and **j** correspond to the parameters *T*_*kill*_, *T*_*min*_, *T*_*OPmin*_, *T*_*OPmax*_, *T*_*max*_, respectively. Dashed lines represent the calibrated parameter values corresponding to each panel.

The distribution of *LL*_*w*_ in *SCE*_*wr*_ had a similar pattern to that in *SCE*_*sr*_ within the corresponding parameter range (Fig. 7). In both scenarios, high *LL*_*w*_ with relatively high density were generally associated with parameter sets close to those in *SCE*_*d*_. Although the maximum *LL*_*w*_ indicates the best fit, its influence on the final outcome was limited due to low density of parameter sets with similarly high likelihood values. In *SCE*_*wr*_, parameter sets that explained the occurrence points poorly had little influence on the final outcome of calibration as their *LL*_*w*_ values approached zero.

The calibrated parameters obtained from *SCE*_*wr*_ were relatively lower than those of *SCE*_*d*_ and *SCE*_*sr*_ (Table 2). For example, the *T*_*min*_ parameter in *SCE*_*wr*_ was 3 ℃ lower than that for *SCE*_*d*_. The differences of the parameter values between *SCE*_*wr*_ and *SCE*_*sr*_ were greater for *T*_*min*_ and *T*_*OPmin*_ than the other parameters. The *LL*_*w*_ value of *SCE*_*wr*_ was greater than those of *SCE*_*sr*_ and *SCE*_*d*_ in the validation set when the 4:1 cross validation was used (see supplementary information 5).

**Table 2:** Calibrated parameter sets obtained from cross validation, along with log-likelihood values (*LL*_*w*_) for the validation set under each parameter scenario. *SCE*_*d*_, *SCE*_*sr*_, *SCE*_*wr*_ represent the default scenario, the calibration scenario with specific range, and the calibration scenario with wide range, respectively. Other symbols are described in Table 1.

Parameter scenario	T_kill_	T_min_	T_OPmin_	T_OPmax_	T_max_	LL_w_
*SCE*_*d*_	0	13	20	30	38	0.836
*SCE*_*sr*_	0.6	10	20.6	30	38.5	0.833
*SCE*_*wr*_	− 1.1	7.8	18.3	29.2	37.8	0.919

### Spatial distribution of climate suitability index

**Fig. 8:**
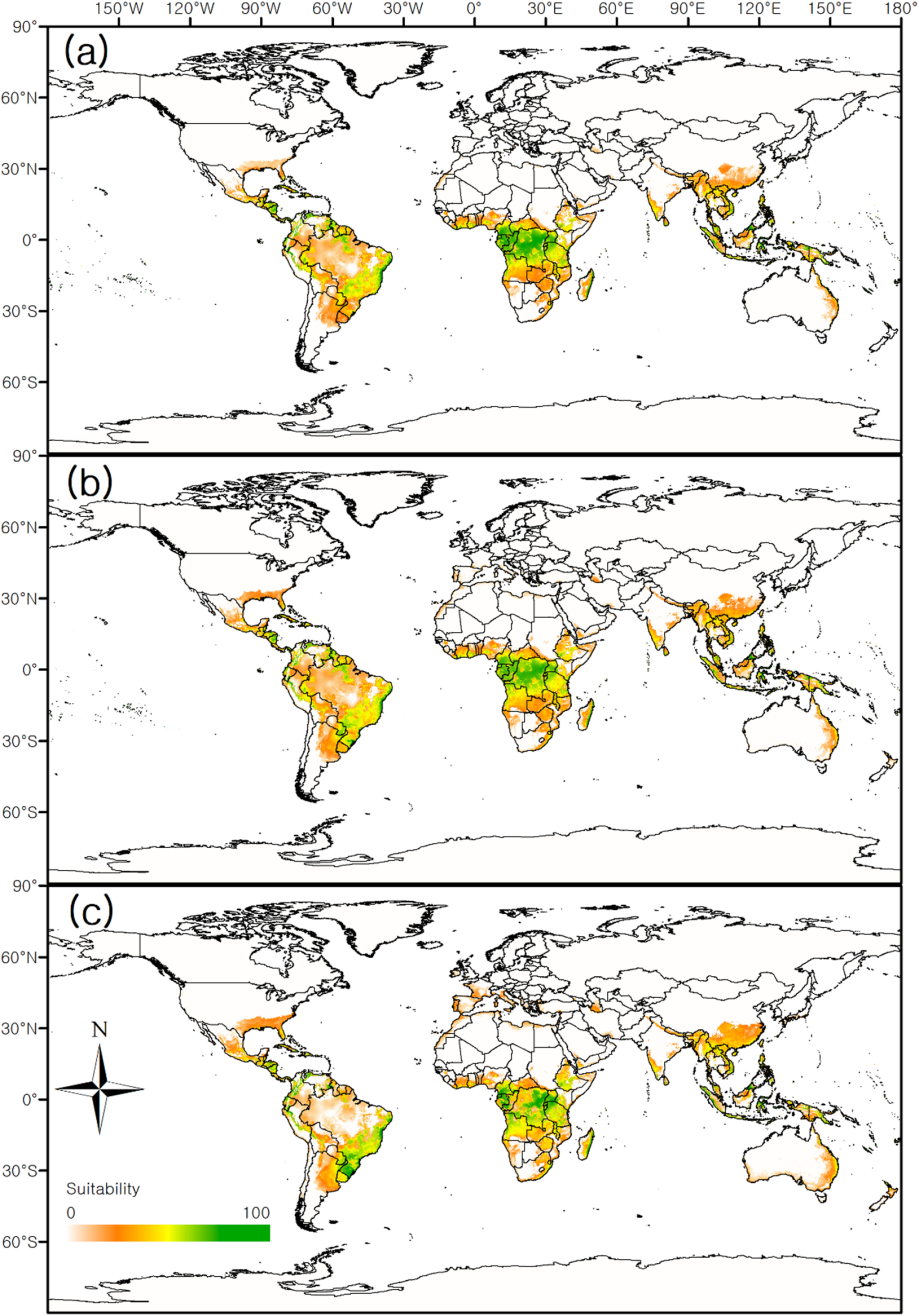
The global climate suitability under **a** the default scenario, **b** the calibration scenario with specific range, and **c** the calibration scenario with wide range. The maps were generated using ArcMap (version 10.5; http://desktop.arcgis.com/en/arcmap/).

The parameter calibration resulted in the spatial pattern of climate suitability values that showed closer correspondence to the distribution of observed orange occurrence sites (Figs. 4 and 8). Both *SCE*_*sr*_ and *SCE*_*wr*_ had higher suitability values than *SCE*_*d*_ for regions with occurrence data, such as Mexico, Spain, and Brazil. Suitability values were also high in other major orange-producing countries such as the United States and China, despite the absence of occurrence data in these countries. In addition, the leading production regions tended to have higher suitability values than the other regions within each country. For example, the mean suitability values were 0.6 and 0.3 for São Paulo in Brazil and Florida in the USA, respectively.

**Fig. 9:**
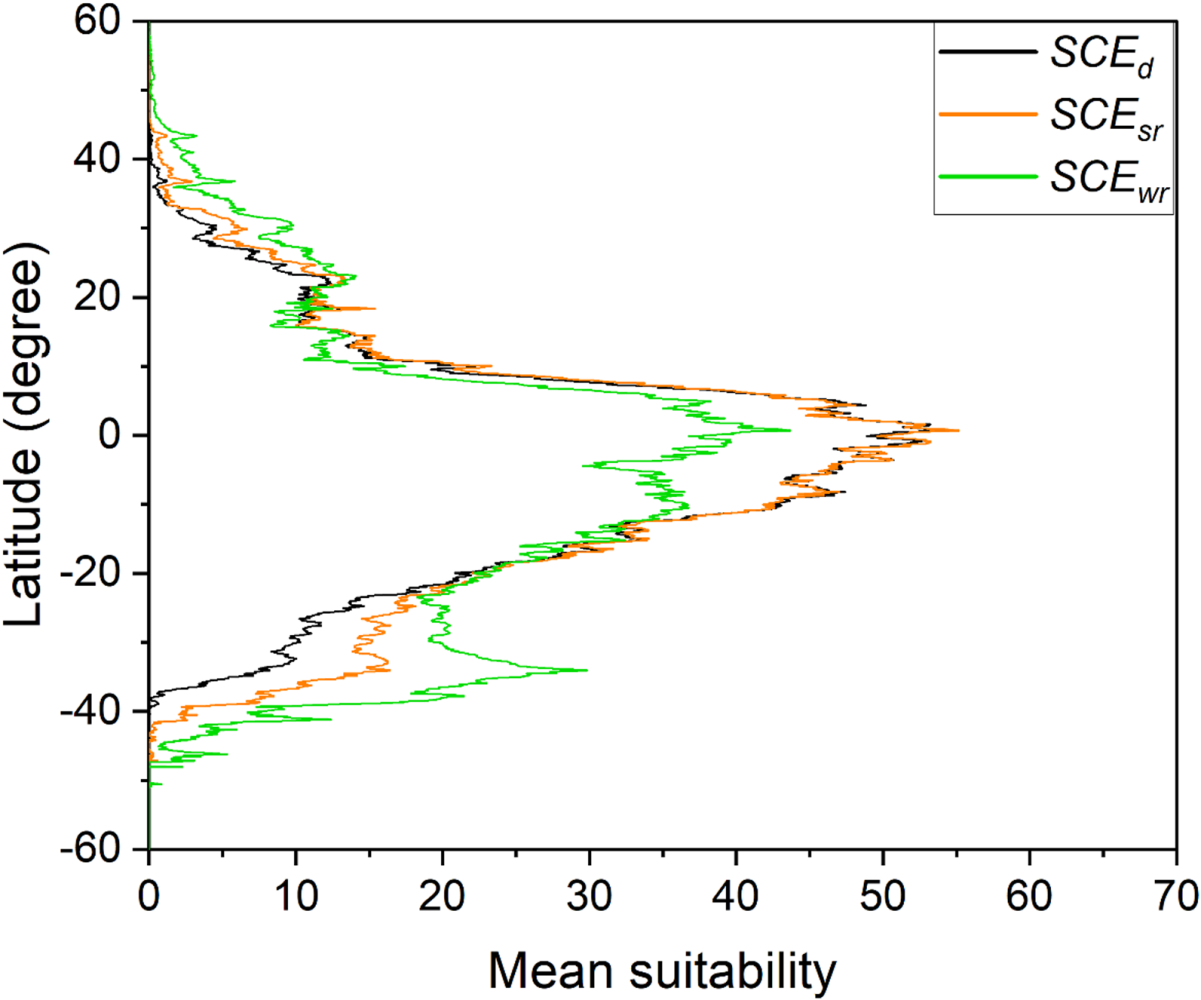
Distribution of suitability values averaged by latitude for the default scenario (*SCE*_*d*_), the calibration scenario with specific range (*SCE*_*sr*_), and the calibration scenario with wide range (*SCE*_*wr*_), respectively.

The mean suitability values by latitude showed a single peak in the low latitude regions, e.g., between 20°S and 20°N, across all scenarios (Fig. 9). Mean suitability values remained positive at higher latitudes up to 40°, a range similar to that of the observed occurrence sites (Fig. 4). Both calibration scenarios had higher mean suitability values than *SCE*_*d*_ between 20°S and 40°S, mainly reflecting higher climate suitability in southern regions of Brazil.

### Comparison between producing and non-producing countries

**Fig. 10:**
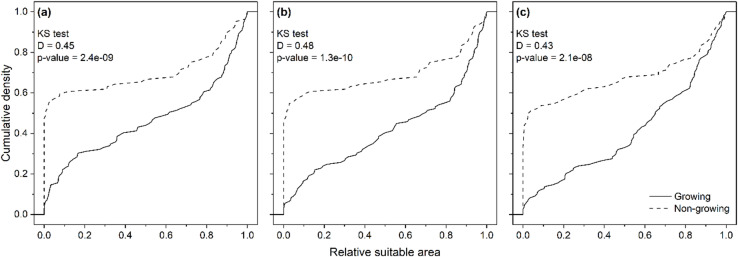
Empirical cumulative distribution functions of relative suitable areas for growing and non-growing countries under **a** default scenario, **b** the calibration scenario with specific range, and **c** the calibration scenario with wide range.

All calibration scenarios were able to differentiate between growing and non-growing countries (Fig. 10), as indicated by the statistically significant separation of their ECDFs (*p* value < 0.01). In all scenarios, the ECDFs of non-growing countries were concentrated at lower *RAs*, whereas those of growing countries were shifted toward higher *RAs*. In particular, *SCE*_*sr*_ showed the largest separation between growing and non-growing countries, with the KS test resulting in a D value of 0.48, compared with 0.45 for *SCE*_*d*_ and 0.43 for *SCE*_*wr*_.

## Discussion

Our results demonstrated that the GLUE method allowed for reasonable calibration of the parameters for the Fuzzy Union model using presence-only data. In particular, reliable results were obtained even with the minimum ecological knowledge of the crop of interest. The use of calibrated parameters tended to result in large areas suitable for oranges in major production countries although no background sample was used. The suitable areas identified using the climate suitability index coincided with those of previous studies^47^. For example, our results agreed with the suitability areas predicted in Sichuan province using the Maxent model^7^. The global distribution map of Asian citrus psyllid (*Diaphorina citri*), which is the major orange pest, was similar to our results^48^. It has been reported that oranges are often produced commercially in countries within tropical, subtropical, and temperate zones, extending to latitudes as far as 40°S^49–51^. Both calibration scenarios resulted in higher climate suitability in areas between 20°S and 40°S in comparison with the default scenario. These suggested that the suitable areas for crop production could be identified reasonably through the calibration using minimum ecological knowledge rather than background data.

The Area Under the Curve (AUC) that requires both presence and absence data has been used to evaluate the reliability of ecological niche models^52^. However, reliable absence data are rarely available^8^ although occurrence records from various databases can be used as presence data. The background data can be generated using various sampling methods and sample sizes to substitute absence data^53^. Still, there is no standard procedure for generation of background data although Phillips et al.^54^ proposed a reasonable approach for selection of background data, e.g., focused on target groups. This would affect the assessment of reliability for the species distribution models^55^. For example, Barbet-Massin et al.^56^ pointed out the lack of consensus on the methods, locations, and quantities of sampling methods for the background samples.

Alternatively, our approach based on the fuzzy logic system facilitated the use of likelihood statistics to compare between presence-only data and model estimates, which required no background data for the calibration of the model. Ecological niche models are often derived from regression models, e.g., generalized linear models^57^, or machine learning models, e.g., Maxent^8^, which require the objective function to organize logical structures for evaluation of climate suitability. In the optimization processes for these models, a pair of presence and absence data would be needed to evaluate the conditional probability of occurrence at sites^54^. In contrast, the Fuzzy Union model already has its logical structures based on the rule statements on evaluation of climate suitability. This facilitated application of a global search algorithm to maximize the likelihood of suitability at occurrence sites in comparison with the entire globe under the assumption that the given species occur within a specific boundary of regions. As a result, no absence data was required for the parameter calibration. This suggested that such an alternative likelihood function could be used to develop a standard procedure for the calibration of species distribution models at the global scale.

Relationships between suitability and local abundance of species have been reported to be triangular or wedge-shaped^58^. Such a relationship forms because areas with a high suitability index would indicate potential habitat rather than the actual cultivation areas, which would be determined by other additional factors^59,60^. Our results suggested that the regions with limited suitability tended to have relatively small cultivation areas whereas those with high suitability did not necessarily coincide with large acreage of oranges. For example, Passos et al.^61^ reported that the citrus production would not be hindered by climate constraints in Brazil, which aligns with our results such that most regions in Brazil were identified to have reasonable climate suitability for orange cultivation. However, the orange production area was about 35% of the total fruit production area in Brazil^42^, suggesting that other crops may be cultivated in the areas suitable for oranges.

Our results indicate that calibration using a wider search space (*SCE*_*wr*_) may be an alternative approach when prior knowledge is insufficient to define appropriate parameter ranges. Although calibration using a specific search range (*SCE*_*sr*_) provided the best performance among the tested scenarios, *SCE*_*wr*_ also showed performance comparable to *SCE*_*sr*_ and *SCE*_*d*_. Similarly, Wu et al.^26^ suggested that calibration outcomes may become unreliable when the search space is defined inappropriately, which is more likely when the background knowledge is limited. This suggests that the proposed calibration framework may be applicable to species with limited knowledge of their climate envelopes, such as invasive plants or neglected and underutilized species (NUS). Still, wide search strategies may increase the risk of equifinality, where different parameter sets result in similar model results^62^. Therefore, the wide-search frameworks should be carefully evaluated in further applications. In this study, however, the risk of equifinality is expected to be relatively small, as the model structure does not exhibit strong interactions among temperature-related parameters or multimodal responses to individual parameters^14^.

One of the limitations in the present study was that only climate conditions were taken into account to determine the spatial suitability of the crop of interest. Actual cultivation would be affected not only by climate but also by biophysical and socio-economic factors^63^. For example, Jaisli et al.^64^ included a wide range of those factors to develop a suitability evaluation system based on a maximum limitation method at regional and global scales. The Fuzzy Union model could be used to substitute the climate suitability within such an integrated assessment system. The fuzzy logic operations could also be expanded to reflect the complexity of crop production and the reliability of climate suitability, although it would be beyond the scope of our study. For example, irrigation management could be applied in addition to rainfall suitability. In the present study, for example, suitability values were low in some orange producing regions such as California and Egypt where irrigation practices play a crucial role in orange production^65^. Irrigation would also have cooling effects^66^, which could reduce the heat stresses in those regions. If irrigation were taken into account, the overall suitability could increase in those regions by compensating for the lack of rainfall (see supplementary information 6).

Further consideration of various physiological processes would be recommended for wider application of the model. For example, the calibration performed for the oranges would also be applicable to other fruit trees located in tropical regions. Still, the current model suffers from the lack of modules to take into account vernalization, which would be required for plants in temperate regions. These plants require an exposure to cold temperature for a certain period to initiate flowering and fruiting^67^. Insufficient chilling accumulation during the cold periods could result in lower yields despite the adequate growing environments^68^. As climate change is reducing the chilling accumulation^69^, the consideration for these impacts would broaden the applicability of the climate suitability model for various temperate fruits.

## Conclusions

This study demonstrated that reliable calibration of climate suitability models was achieved using the GLUE method with presence-only data. Through a case study for oranges, we found that the use of a weighted likelihood function contributed to a more robust distinction between reliable and unreliable parameter sets. Furthermore, the calibrated parameters, particularly those obtained using wide search space, improved the prediction accuracy of suitable regions compared to the default parameter set. This approach shows potential for broader application across different crops, even for those with limited background knowledge. It would be especially useful for species such as invasive plants or neglected and underutilized species (NUS), where occurrence data are available but ecological parameters are poorly defined. Consequently, our findings suggest that this method could be utilized as an alternative tool in addressing shifts in cultivation areas due to climate change and supporting agricultural land use planning.

## Supplementary Information

Below is the link to the electronic supplementary material.

Supplementary Material 1

## Data Availability

All of the material is owned by the authors and/or no permissions are required.The datasets used and/or analyzed during the current study available from the corresponding author on reasonable request.

## Declarations

### Generative AI and AI-assisted technologies

During the preparation of this work the authors used chatGPT in order to improve readability and language. After using this tool/service, the authors reviewed and edited the content as needed and take full responsibility for the content of the publication.
