# Supplementary material for: Calibration of a climate suitability model using a generalized likelihood uncertainty estimation (GLUE): a global case study of orange production
Source: Sci Rep. 2026 Apr 17;16:18287. doi: 10.1038/s41598-026-44664-5 (PMC13261111; doi:10.1038/s41598-026-44664-5)
Supplement: Supplementary file 1 — Supplementary Material 1 [file 41598_2026_44664_MOESM1_ESM.docx]

Calibration of a Climate Suitability Model Using a Generalized Likelihood Uncertainty Estimation (GLUE): A Global Case Study of Orange Production

Shinwoo Hyun, Kwang Soo Kim, Robert M. Beresford

Supplementary information 1. Membership functions of the Fuzzy Union model

The Fuzzy Union model evaluates a set of rules for climate conditions, which are defined using linguistic terms such as a statement “temperature is suitable”. In the model, the degree of truth is determined for the rule statements associated with the climate envelopes, e.g., maximum and minimum ranges of temperature and precipitation using the membership functions (figure S1). For example, temperature suitability is assessed using the length of time periods during which a range of optimum temperatures occurred in the climate suitability model. The outcomes of the rule statements are determined for each month.

A suitability index for a start month *s* and growing period *G* (*S_s,G_*) is calculated by averaging monthly suitability indices as follows:

$$\begin{aligned} S_{s,G}=\sum_{m=1}^{G} \left( \theta_{m}+\beta_{m}-\theta_{m}\cdot\beta_{m}-\tau_{m} \right)\cdot\prod_{m=1}^{G} \left( 1-\kappa_{m} \right)\cdot\frac{100}{G} \#\left( equation S1 \right), \end{aligned}$$

where, $\theta_{m}$, $\beta_{m}$, $\tau_{m}$, $\kappa_{m}$ represent the suitability for temperature and precipitation, the stress index, and the harmful index for *m-*th month from the planting, respectively. The final suitability (S) is determined by selecting median across the possible growing periods and the maximum across all the start months in a year as follows:

$$\begin{aligned} S=\max\left\{ median\left\{ S_{s,G} | G_{min}\leq G\leq G_{max} \right\} | 1\leq s\leq12 \right\} \#\left( equation S2 \right). \end{aligned}$$

Detailed description on the Fuzzy Union model can be found in Kim et al. (2018).


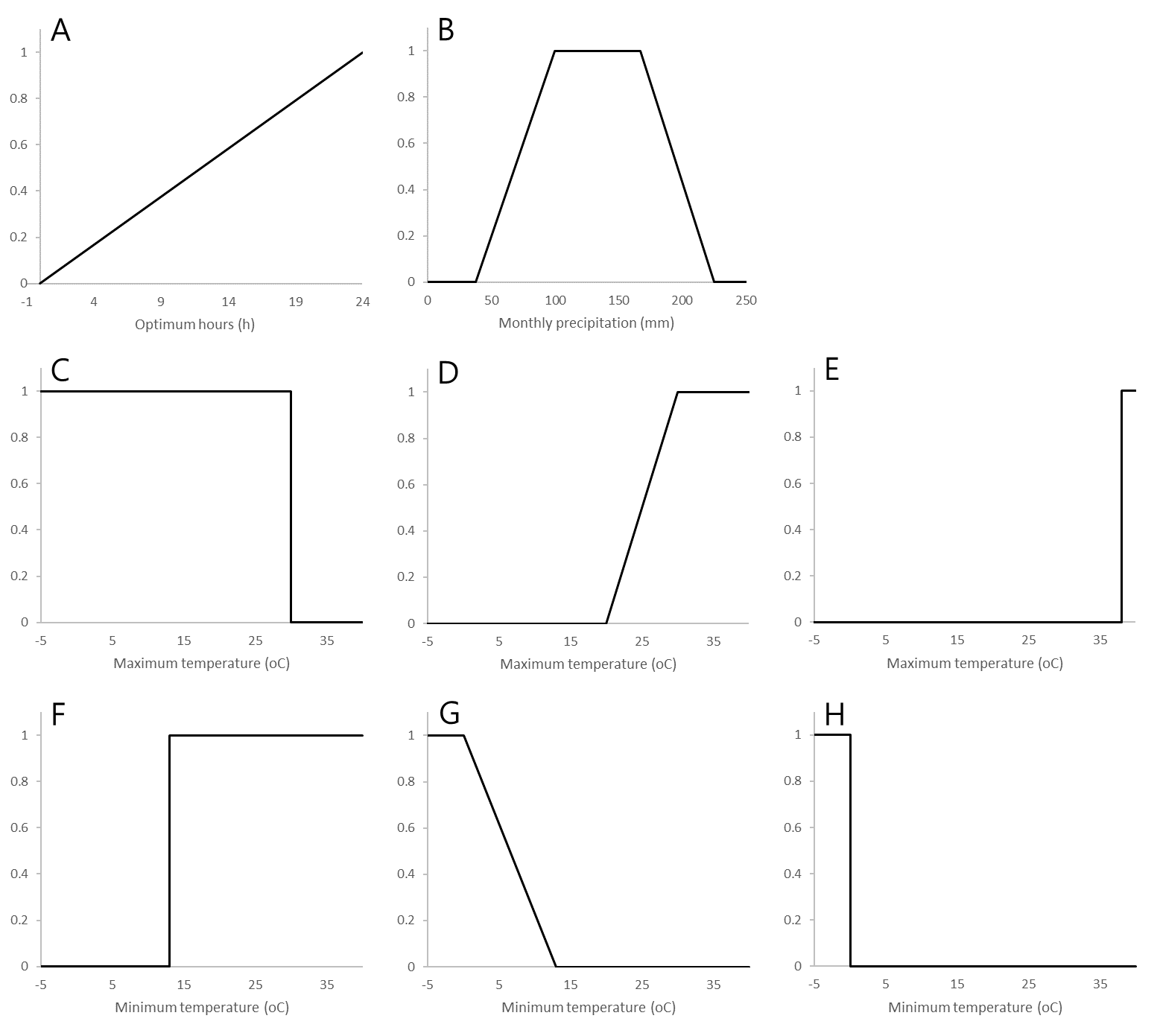


Figure S1. Membership functions of the Fuzzy Union model (Kim et al., 2018). The membership functions represent (A, B) suitable, (C, F) favorable, (D, G) stressful, and (E, H) harmful conditions. They describe responses to (A) optimum temperature duration, (B) monthly precipitation, (C, D, E) maximum temperature, and (F, G, H) minimum temperature.

Supplementary information 2. GLUE procedures for parameter calibration

1. Generation of random parameter sets from prior distribution: The random parameter sets were sampled from the uniform distribution with lower and upper bounds (He et al. 2009; Bian et al. 2023). In the present study, Sobol sequence was used to generate points that are evenly distributed across the parameter space (Renardy et al. 2021). This ensured that all the regions of the search space were sampled evenly, avoiding clustering of points in some areas or gaps in others, which would improve the efficiency and accuracy of parameter space exploration (Burhenne et al. 2011).
2. Operation of the model: The climate suitability model was executed using each parameter set to generate a climate suitability map.
3. Calculation of likelihood and probability values for each parameter set: Likelihood values were calculated by evaluating the similarity between the climate suitability map produced by the model and the observed occurrence sites. Then, the probability of each parameter set was computed with the following equation:

$$\begin{aligned} p\left( {PRM}_{i} \right)=\frac{L\left( PRM_{i} | OBS \right)}{\sum_{i=1}^{N} L\left( PRM_{i} | OBS \right)} \#\left( equation S3 \right), \end{aligned}$$

where *p(PRM_i_)* is the probability value of the *i* th parameter set *PRM_i_*, and *L(PRM_i_|OBS)* is the likelihood value of the *PRM_i_* given occurrence points *OBS*. *N* represents the number of random parameter sets generated in the first step.
4. Determination of parameter value from posterior distribution: Mean and variance of posterior distribution for each parameter are calculated using the pairs, (*PRM_i_, p_i_*), *i* = 1, …, *N*, with the following equation:

$$\begin{aligned} \mu\left( PRM \right)=\sum_{i=1}^{N} p\left( PRM_{i} \right)\cdot PRM_{i} \#\left( equation S4 \right), \end{aligned}$$

$$\begin{aligned} \sigma^{2}\left( PRM \right)=\sum_{i=1}^{N} p\left( PRM_{i} \right)\cdot\left( PRM_{i}-\mu\left( PRM \right) \right)^{2} \#\left( equation S5 \right), \end{aligned}$$

where $\mu(PRM)$ and $\sigma^{2}(PRM)$ represent the estimated mean and variation of the posterior distribution of parameter *PRM*, respectively.

The threshold value of *TH* was determined to be the 10^th^ percentile of the climate suitability index at the occurrence sites in the calibration dataset (Barredo et al. 2015; Kramer-Schadt et al. 2013).

Supplementary information 3. Likelihood values using the existing method

The final values of *LL_Kim_* could become identical for two different sets of suitability index (figure S2). The changes in the empirical cumulative distribution functions of suitability at the occurrence sites (*E_o_(i) -E_o_(i-1))* and across the entire region (*E_a_(i)- E_a_(i-1)*) were used to compute the value of log ratio (LR; figures S2A-B). Subsequently, the cumulative sums (CS) of the LR values were determined, yielding identical results across the data sets (figures S2C-D).


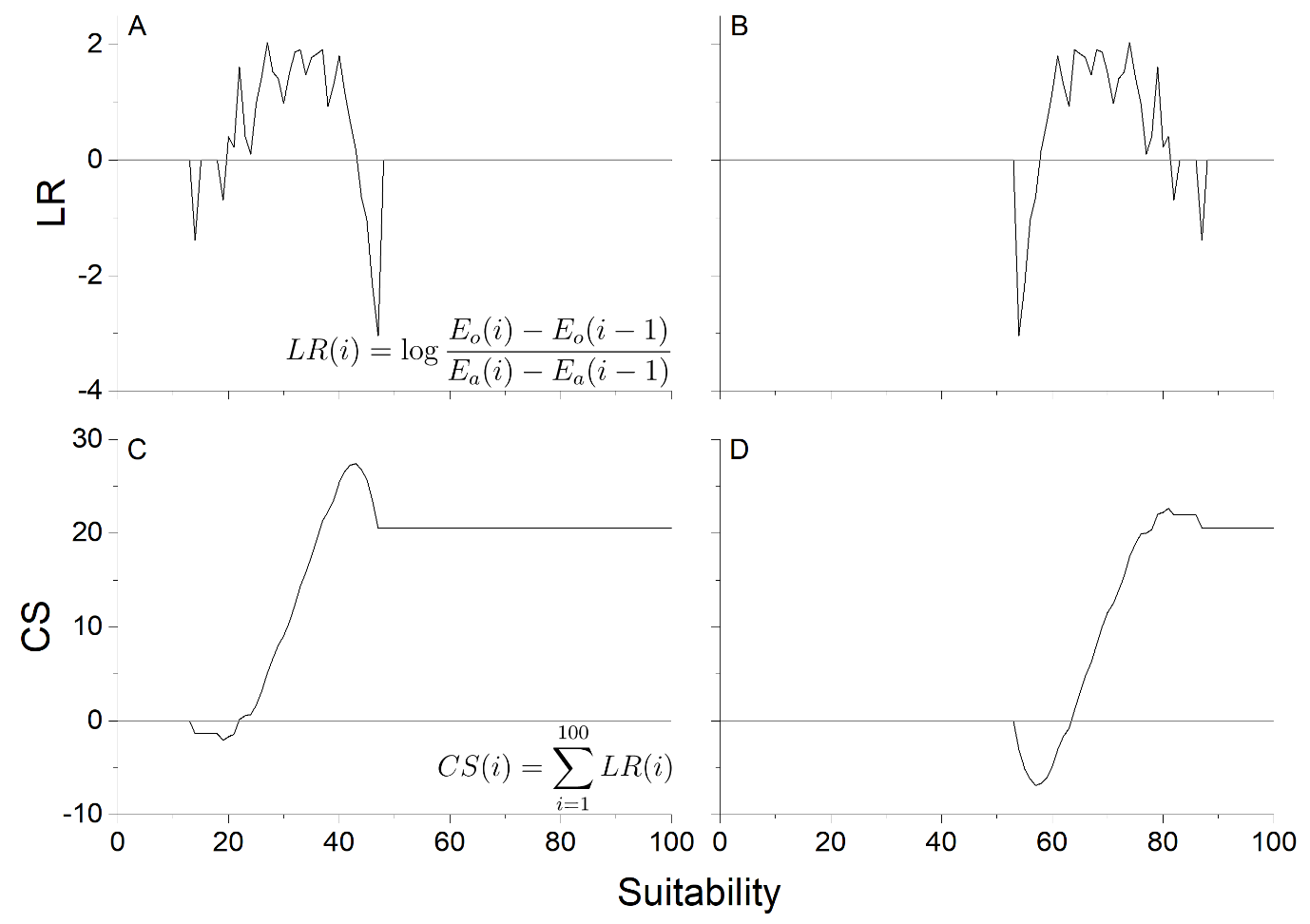


Figure S2. (A, B) Log ratios between the differences in empirical cumulative density functions of suitability values at occurrence sites and those across the entire region, and (C, D) their cumulative sum. The mean value of climate suitability at the occurrence sites is higher than that in the region (A and C) and vice versa (B and D).

Supplementary information 4. Search space of parameters.

The EcoCrop database was used as a source of existing knowledge for orange cultivation. Accordingly, the parameter search space for the calibration scenario with specific ranges (*SCE_sr_*) was determined based on the values reported in the database (Table 1). The lower and upper bounds of each parameter were determined using related parameters reported in the database. For example, the range of the *T_min_* parameter was set using the values of *T_kill_* and *T_OPmin_* from the database. The upper bound of the *T_max_* parameter was defined by adding the difference between *T_OPmax_* and *T_max_* values to the existing *T_max_* value. In addition, the lower bound of the *T_kill_* parameter was determined using the killing temperature during the rest period. These settings ensure that the parameter values are constrained within reasonable ranges based on existing knowledge.

An alternative calibration approach was applied using a wide range of search space, *SCE_wr_*, under the assumption of limited knowledge for the given species. This scenario involved setting the parameter search space by sampling temperature parameters within the range from -10 to 46 ℃ to make the least use of the EcoCrop database.

Supplementary information 5. Calibration using existing likelihood function

Parameters for oranges were calibrated using the existing likelihood function (*L_Kim_*) proposed by Kim et al. (2018) in addition to using the weighted likelihood (*L_w_*) (Table S1). The parameter values were considerably different from the default values, which caused lower likelihood values for both *SCE_sr_* and *SCE_wr_*. This resulted from the extremely large values of likelihood for a small number of parameter sets when *LL_Kim_* and *L_Kim_* were used for calibration in comparison with the weighted method, *LL_w_* and *L_w_* (figures S3-S4).

Table S1. Calibrated parameter sets obtained from cross-validation, along with log-likelihood values (*LL_Kim_*) for the validation set under each parameter scenario.

| Parameter scenario | *T_kill_* | *T_min_* | *T_OPmin_* | *T_OPmax_* | *T_max_* | *LL_Kim_* | *LL_w_* |
| --- | --- | --- | --- | --- | --- | --- | --- |
| *SCE_d_* | 0 | 13 | 20 | 30 | 38 | 117.4 | 0.836 |
| *SCE_sr_* using *LL_Kim_* | 2.8 | 10.4 | 14.2 | 25 | 42.5 | 51.6 |  |
| *SCE_wr_* using *LL_Kim_* | 4.7 | 9.0 | 14.0 | 25.7 | 40.7 | 57.2 |  |
| *SCE_sr_* using *LL_w_* | 0.6 | 10 | 20.6 | 30 | 38.5 |  | 0.833 |
| *SCE_wr_* using *LL_w_* | -1.1 | 7.8 | 18.3 | 29.2 | 37.8 |  | 0.919 |


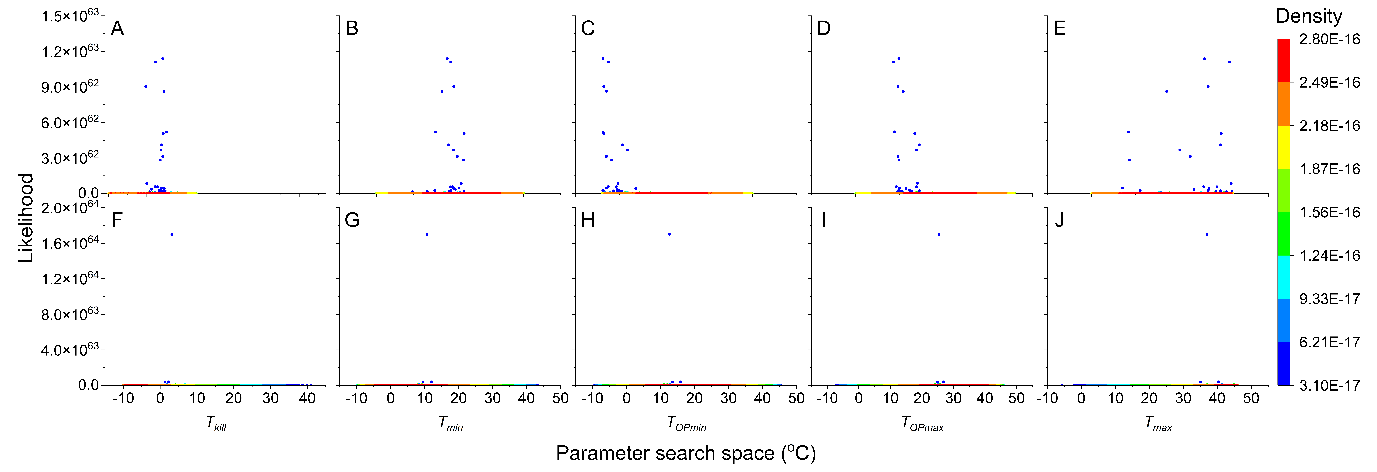


Figure S3. Distribution of likelihood values (*L_Kim_*) for the parameter sets obtained from the calibration, shown by parameter and search space. Panels A to E and F to J represent *L_Kim_* distributions under the specific range calibration scenario (*SCE_sr_*), and wide range calibration scenario (*SCE_wr_*), respectively. Panel pairs A and F, B and G, C and H, D and I, and E and J correspond to the parameters *T_kill_*, *T_min_*, *T_OPmin_*, *T_OPmax_*, *T_max_*, respectively.


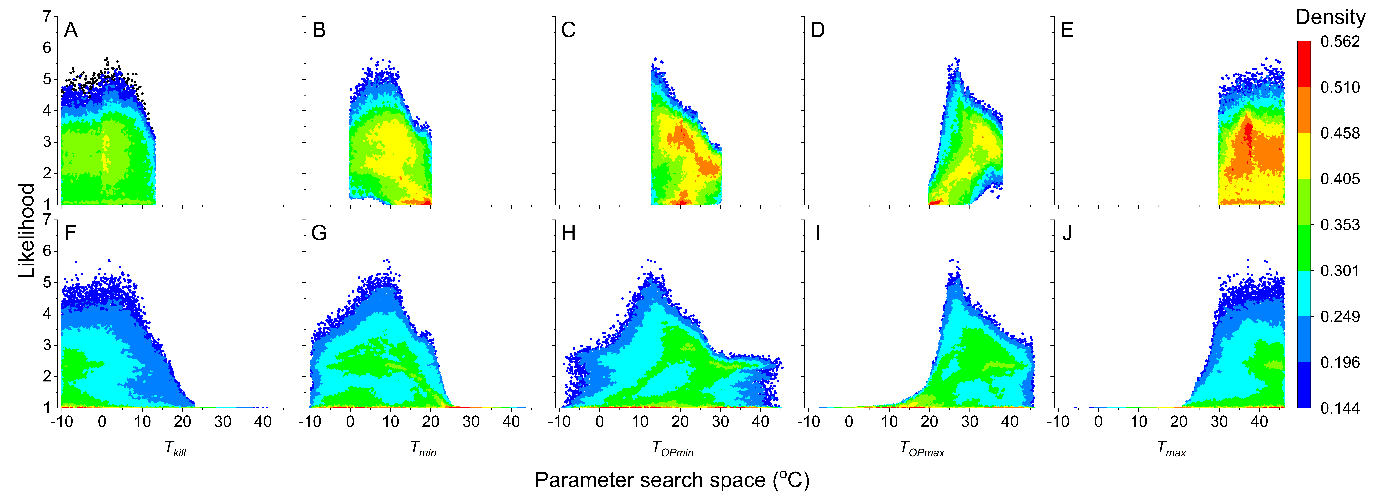


Figure S4. Distribution of weighted likelihood values (*L_w_*) for the parameter sets obtained from the calibration, shown by parameter and search space. Panels A to E and F to J represent *L_w_* distributions under the specific range calibration scenario (*SCE_sr_*), and wide range calibration scenario (*SCE_wr_*), respectively. Panel pairs A and F, B and G, C and H, D and I, and E and J correspond to the parameters *T_kill_*, *T_min_*, *T_OPmin_*, *T_OPmax_*, *T_max_*, respectively.

Supplementary information 6. Climate conditions for regions where irrigation is required

Monthly temperature and precipitation data were extracted at four sites in arid regions in California, the United States (119.4179°W, 36.7783°N), Al-Dakahlia, Egypt (31.24967°E, 30.8025°N), Yucatan, Mexico (89°W, 20.5°N), and Valencia, Spain (0.5°W, 39°N). The climate conditions were compared with the parameter values from *SCE_wr_* (figure S5). These sites had low values of suitability index due to low precipitation and heat stresses.


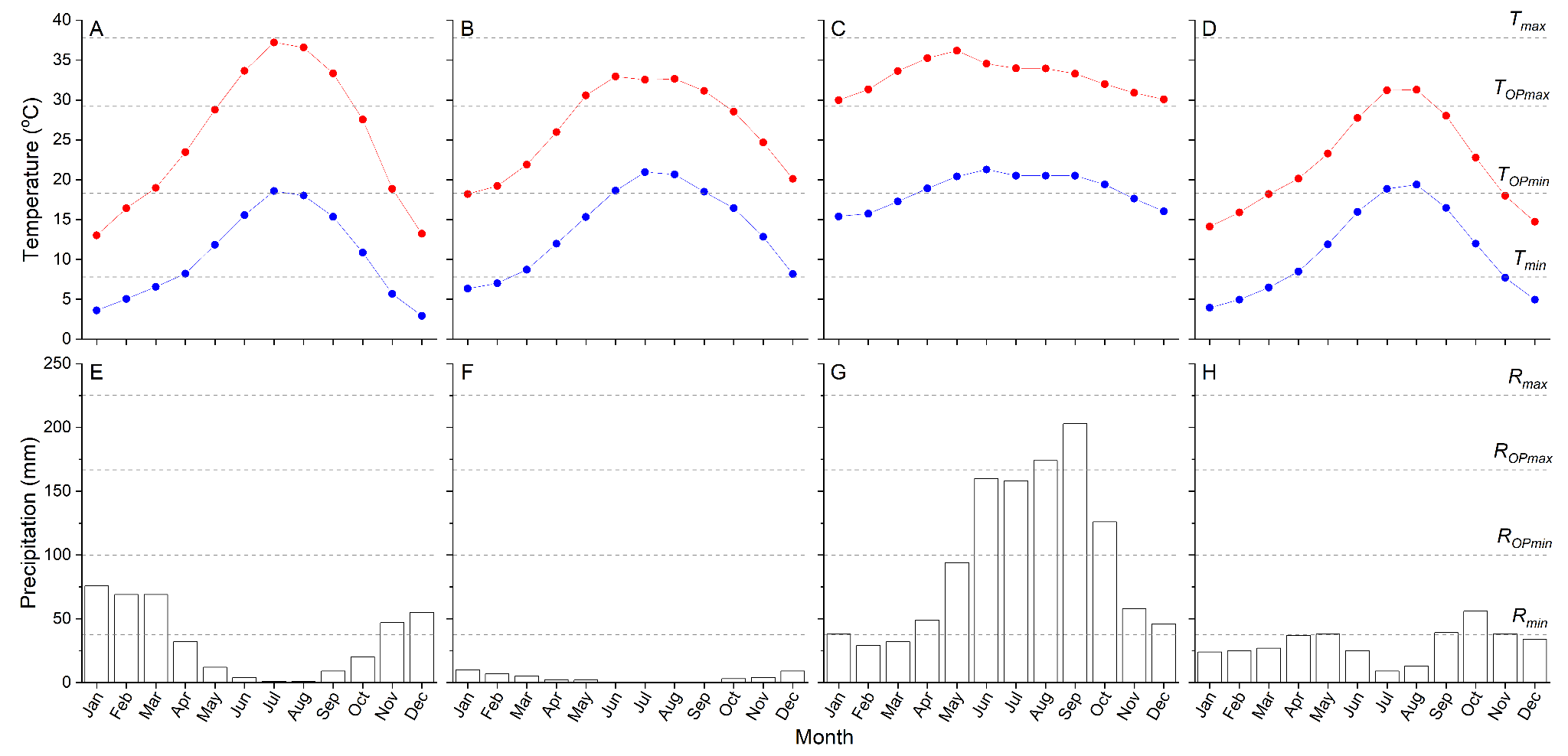


Figure S5. Climate conditions in (A, E) California, United States (B, F) Al-Dakahlia, Egypt (C, G) Yucatan, Mexico and (D, H) Valencia, Spain. These conditions include (A-D) monthly temperature and (E-H) precipitation. The parameters for temperature and precipitation are indicated on the y axis.

Reference

Barredo, J., Strona, G., de Rigo, D., Caudullo, G., Stancanelli, G., San‐Miguel‐Ayanz, J., 2015. Assessing the potential distribution of insect pests: case studies on large pine weevil (Hylobius abietis L) and horse‐chestnut leaf miner (Cameraria ohridella) under present and future climate conditions in European forests. EPPO Bulletin 45, 273-281.

Bian, J., Wu, J., Nie, S., Wang, Y., Lin, X., 2023. The parametric uncertainty estimation of water and nitrogen transport simulation in a paddy field experiment using HYDRUS-1D. Irrigation and Drainage, 1-14.

Burhenne, S., Jacob, D., Henze, G.P., 2011. Sampling based on Sobol’ sequences for Monte Carlo techniques applied to building simulations, Proc. Int. Conf. Build. Simulat, pp. 1816-1823.

He, J., Dukes, M.D., Jones, J.W., Graham, W.D., Judge, J., 2009. Applying GLUE for estimating CERES-Maize genetic and soil parameters for sweet corn production. Transactions of the ASABE 52, 1907-1921.

Kim, H., Hyun, S.W., Hoogenboom, G., Porter, C.H., Kim, K.S., 2018. Fuzzy Union to Assess Climate Suitability of Annual Ryegrass (Lolium multiflorum), Alfalfa (Medicago sativa) and Sorghum (Sorghum bicolor). Sci. Rep. 8, 10220.

Kramer‐Schadt, S., Niedballa, J., Pilgrim, J.D., Schröder, B., Lindenborn, J., Reinfelder, V., Stillfried, M., Heckmann, I., Scharf, A.K., Augeri, D.M., 2013. The importance of correcting for sampling bias in MaxEnt species distribution models. Divers. Distrib. 19, 1366-1379.

Renardy, M., Joslyn, L.R., Millar, J.A., Kirschner, D.E., 2021. To Sobol or not to Sobol? The effects of sampling schemes in systems biology applications. Math. Biosci. 337, 108593.
